# New 3*H*-Indole Synthesis by Fischer’s Method. Part I

**DOI:** 10.3390/molecules15042491

**Published:** 2010-04-08

**Authors:** Sami Sajjadifar, Hooshang Vahedi, Abdolhossien Massoudi, Omid Louie

**Affiliations:** 1 Department of Chemistry, Payame Noor University (PNU), P O Box 91735-433, Mashhad, Iran; E-Mails: hooshangvahedi@yahoo.co.uk (H.V.); Massoudi_h@pnu.ac.ir (A.M.); o_louie2001@yahoo.com (O.L.); 2 Department of Chemistry, Payame Noor University (PNU), Ilam, Iran

**Keywords:** 3H-indole, indolenine, Fischer’s synthesis method, acetic acid

## Abstract

Methyl indolenines (**4a-c**) and (**5a-c**) were prepared in high yield by a Fischer indole synthesis reaction of *o,m*-tolylhydrazine hydrochlorides (**1a-b**) with isopropyl methyl ketone (**2**) and 2-methylcyclohexanone (**3**) in acetic acid at room temperature. *o,p*- Nitrophenylhydrazines (**1c-d**) were reacted with 2-methylcyclohexanone (**3**) in acetic acid at reflux to give nitroindolenines (**5d-e**), while the attempted reactions of *o,p*-nitrohydrazines with isopropyl methyl ketone (**2**) in acetic acid were not successful. Compounds (**1c-d**) were reacted with isopropyl methyl ketone (**2**) in acetic acid/HCl to give 2,3,3-trimethyl-5-nitro-indolenine (**4e**) and 2,3,3-trimethyl-7-nitroindolenine (**4d**).

## Introduction

The Fischer indole synthesis is a chemical reaction that produces an aromatic heterocyclic indole from a (substituted) phenylhydrazine and an aldehyde or ketone under acidic conditions. The reaction was discovered in 1883 by Hermann Emil Fischer [1]. Today antimigraine drugs of the tryptan class are often synthesized by this method. The choice of acid catalyst is very important. Bronsted acids such as HCl, H_2_SO_4_, polyphosphoric acid and *p*-toluenesulfonic acid have been used successfully, while. Lewis acids such as boron trifluoride, zinc chloride, iron chloride, and aluminium chloride are also useful catalysts [2,3,4].

**Scheme 1:**
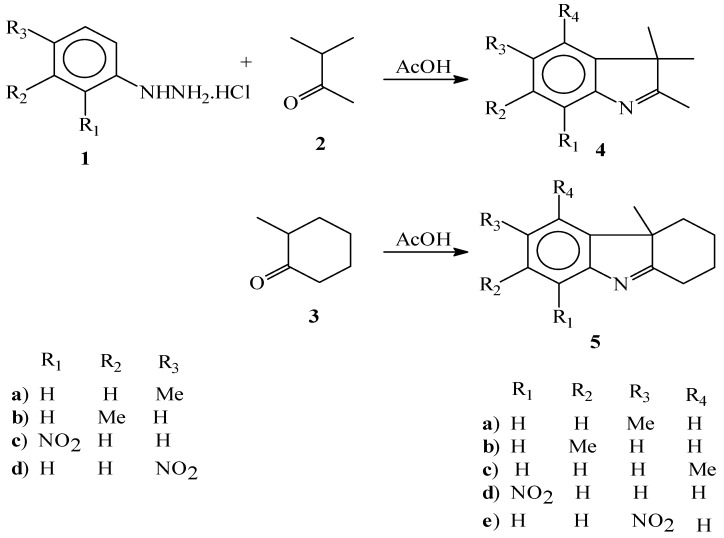
The Fischer reaction for indolenine synthesis.

The reaction of a (substituted) phenylhydrazine with an aldehyde or ketone initially forms a phenylhydrazone which isomerizes to the respective enamine (or 'ene-hydrazine'). After protonation, a cyclic [3,3]-sigmatropic rearrangement occurs producing an imine. The resulting imine forms a cyclic aminoacetal (or aminal), which under acid catalysis that eliminates NH_3_, resulting in the energetically favorable aromatic indole. The accepted mechanism for Fischer’s synthesis was suggested by Robinson [2,3]. According to Robinson mechanism, this reaction has three steps: (a) tautomeric conversion of hydrazone to enehydrazine, (b) carbon-carbon bond formation, (c) cyclization with ammonia, elimination and finally indole synthesis. Some researchers [5] believed that the carbon-carbon bond formation step is the most important and rate-determining step. This step can control the amount of indoles. The presence of different ketones for indole synthesis has been investigated. Isotopic labeling studies show that the aryl nitrogen (N1) of the starting phenylhydrazine is incorporated into the resulting indole [6,7] Scheme 1.

## Results and Discussion

As mentioned in the Introduction, the use of different ketones for indole synthesis has been investigated, but so far, the effects of substituent groups on the phenyl hydrazine benzene ring on the Fischer indole synthesis have not been reported. Therefore, the presence of methyl and nitro groups in different phenyl hydrazines and the use of isopropyl methyl ketone (**2**) and 2-methylcyclohexanone (**3**) resulted in a new synthesis of indolenines in which the yield significantly high, mainly a single product is obtained and it can be carried out under simple conditions. 

^1^H-NMR spectrum of indolenine (**4a**) shows a singlet at δ = 1.16 ppm corresponding to the two methyl groups at C-3, a singlet methyl group for C-2 at δ = 2.24 ppm, and a methyl group singlet resonance for Ar-CH_3_ at δ = 2.1 ppm. In the UV spectrum of (**4a**), two absorption bands of 217 and 262 nm were seen. After acid addition, position shifts in the band maxima was noticed which are the result of presence of the non-bonding pair electrons in the H^+^ reaction. Moreover, the presence of the donating electron methyl groups in suitable positions supported this bathochromic effect. 

*m*-Tolylhydrazine hydrochloride (**1b**) reacted at room temperature with isopropyl methyl ketone (**2**) at the presence of glacial acetic acid to afford two isomers - 2,3,3,4-tetramethylindolenine (**4c**) and 2,3,3,6-tetramethylindolenine (**4b**) - in 88% yield. Our efforts to separate the two isomers through TLC and column chromatography in different solvents were fruitless. 

The^ 1^H-NMR spectrum of the two isomers displays two singlets at δ = 1.25 and δ = 1.35 ppm corresponding to the C-3 methyl hydrogens, two methyl group of C-2 at δ = 2.33 and δ = 2.37 ppm, a Ar-CH_3_ methyl group singlet at δ = 2.19 and in the aromatic region, a doublet at δ = 6.84 ppm and a singlet at δ = 7.1. In the IR spectrum, a C=N stretching vibration is seen at 1,700 cm^-1^. In the UV spectrum of this mixture, two absorption bands at 218 and 259 nm were seen and a bathochromic effect was noticed after acid addition. 

The ^1^H-NMR spectrum of indolenine (**5a**) showed a methyl group singlet at δ = 1.15 ppm which provides strong support for the cyclization and creation of indolenine (**5a**). In the IR spectrum, a C=N stretching vibration was observed at 1,700 cm^-1^, which indicates the absence of the N-H bond. In the UV spectrum of (**5a**) two absorption bands at 212 and 263 nm were observed, which after acid addition shifted position. This indicates the presence of non-bonding pair electrons in the reaction with H^+^. 

*m*-Tolylhydrazine hydrochloride (**1b**) was also reacted at room temperature with 2-methyl-cyclohexanone (**3**) in the presence of glacial acetic acid. After extraction, the products were passed through a short silica gel column. Two isomers, 4a,5- and 4a,7-dimethyl-1,2,3,4-tetrahydro-4a*H*-carbazole (**5b-c**), were obtained. The efforts to separate these products were fruitless. The ^1^H-NMR spectrum of the mixture of isomers indicated two singlet for the C-4a methyl groups at δ = 1.14 and **δ** = 1.24, which proves the cyclization and formtion of the indolenines (**5b-c**). The singlet signals of the methyl groups in the two isomers overlap at δ = 2.21. In the IR spectrum the C=N stretching vibration was observed at 1,695 cm^-1^. In the UV spectrum the maximum absorptions were noticed at 214 and 259 nm which after acid addition, a shift in the position of the band maxima was also seen, which is indicative of the presence of non-bonding pair electrons in the reaction with H^+ ^(bathochromic effect). 

*p*-Nitrophenylhydrazine hydrochloride (**1d**) and isopropyl methyl ketone (**2**) were reacted in the presence of acetic acid and the mixture was refluxed for 1.5 h. The product, 2,3,3-trimethyl-5-nitroindolenine (**4d**), was obtained in very low yield (10%). During the long reaction under reflux conditions, the instability and sensitivity of the products led to their decomposition, oxidation and polymerization. The reaction was also carried out in a binary mixture of acetic acid and hydrochloric acid for 4 h. After extraction, the product (**4e**) was obtained in 30 % yield. The ^1^H-NMR spectrum of indolenine (**4e**) showed a singlet signal for the two C-3 methyl groups at δ = 1.35 and a methyl group singlet for C-2 at δ = 1.87. In the aromatic region, three hydrogens were observed. 

## Conclusions

It is shown that by switching from –OMe to –NO_2_, either the time of the reaction will be longer or reaction conditions will be harsher, so when there is an electron-withdrawing group on the benzene ring in the phenylhydrazine, the efficiency is lower and the reactions are much more difficult. This research demonstrates that Robinson’s proposed mechanism is correct. The effects of the studied groups in the indolenine formation reaction are summarized in Table 1, Table 2.

**Table 1:** Reactions with isopropyl methyl ketone (**2**). 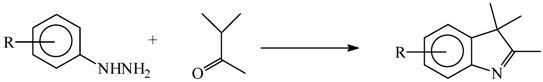

R	Condition	Time	Acid	Y %
**p-OMe**	RT	20 min	AcOH	85
**p,o-OMe**	RT	2 h	AcOH	>90
**o,m,p-Cl**	reflux	0.5 h	AcOH	>90
**o,p-NO_2_**	reflux	90 h	AcOH	9
		4 h	AcOH, HCl	31

**Table 2:** Reactions with 2-methylcyclohexanone (**3**). 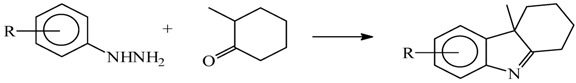

R	Condition	Time	Acid	Y %
**p-OMe**	RT	20 min	AcOH	>90
**p,o-OMe**	RT	1.5 h	AcOH	>90
**o,m,p-Cl**	reflux	0.5 h	AcOH	>90
**o,p-NO_2_**	reflux	24 h	AcOH	53

## Experimental

### General

Melting points were recorded on a Philip Harris C4954718 apparatus and are uncorrected. ^1^H- and ^13^C-NMR spectra were recorded on a Bruker Avance AQS 75 MHz spectrometer. Chemical shifts δ are in parts per million (ppm) measured in CDCl_3_ as solvent and relative to TMS as the internal standard. Infrared spectra were recorded on a Thermo Nicolet-Nexus 670 FTIR instrument and elemental analyses were carried out on an Exeter analytical model CE440 C, H and N elemental analyzer.

### 2,3,3,5-Tetramethylindolenine (**4a**)

*p*-Tolylhydrazine hydrochloride (**1a**, 0.25 g, 1.62 mmol) and isopropyl methyl ketone (**3**, 0.14 g, 1.62 mmol) were added to glacial acetic acid (2 g, 0.03 mol). The mixture was refluxed for 2.25 h with stirring. TLC indicated the end of the reaction and formation of the product. The mixture was cooled and neutralized with 1 M NaOH then diluted with water (100 mL) and extracted with CDCl_3_ (3 × 100 mL). Following the drying of the organic layer over Na_2_SO_4_, the solvent was removed by means of evaporation and the residue was passed through a short silica gel column for further purification. A red, viscous oil of 2,3,3,5-tetramethylindolenine (**4a**) was obtained. TLC: R_F_ 0.22 on silica gel eluting with PhCH_3_-EtOAc (9:1); UV: (EtOH) λ_max_ (nm) 217 (H, 2.80) 262 (H, 1.557), (EtOH, HCl) 207 (H, 1.706), 236 (H, 1.487), 289 (H, 1.310); IR (cm^-1^): 2940, 1680, 1570, 1450, 1363, 1190, 810; ^1^H-NMR δ (ppm): 1.16 (s, 6H, 2×CH_3_), 2.1 (s, 3H, Ar-CH_3_), 2.24 (s, 3H, N=C-CH_3_), 6.7-6.9 (b, 1H, Ar-H), 6.87 (s, 1H, Ar-H), 7.1-7.2 (b, 1H, Ar-H); ^13^C-NMR δ (ppm): 19.9, 24.6, 25.1, 37.8, 121.9, 127.6, 129.3, 136.6, 148.8, 152.7, 164.6; C_12_H_15_N, Mol. Wt: 173.2542; Elem. Anal.: C, 83.19; H, 8.73; N, 8.08.

### Reaction of (**1b**) with ketone (**2**) to produce indolenines (**4b**) and (**4c**)

*m*-Tolylphenylhydrazine hydrochloride (**1b**, 0.3 g, 1.89 mmol) and isopropyl methyl ketone (**2,** 0.17 g, 1.98 mmol) were added to glacial acetic acid (3 g, 0.05 mol) and stirred for 2 h. TLC indicated the end of reaction and formation of the product. The mixture was then cooled, neutralized with 1 M NaOH, diluted with water (100 mL) and finally extracted with CDCl_3_ (3 × 100 mL). Following the drying of the organic layer over Na_2_SO_4_, the solvent was removed by evaporation and the residue passed through a short column of silica gel for further purification. Attempts to separate the two isomers were not successful, but one the of isomers [2,3,3,6-tetramethylindolenine (**4b**)] was isolated and separated by preparative TLC eluting with CH_2_Cl_2_-CH_3_COCH_3_ (20:1) and the data of another isomer inferred by comparing with the data of the two isomer mixture.

*2,3,3,6-Tetramethylindolenine *(**4b**): TLC: R_F_ 0.3 on silica gel eluting with CH_2_Cl_2_-CH_3_COCH_3_ (20:1); UV: (EtOH) λ_max_ (nm) 219 (H, 2.292) 261 (H, 0.684), (EtOH, HCl) 210 (H, 1.513) 227 (H, 1.088) 280 (H, 0.625); IR (cm^-1^): 2900, 1700, 1600, 1565, 1450, 1425, 1340, 1250, 1130, 800; ^1^H-NMR: 1.25 (s, 6H, 2×CH_3_), 2.19 (s, 3H, Ar-CH_3_), 2.33 (s, 3H, N=C-CH_3_), 6.7-6.9 (b, 2H, Ar-H), 7.1 (s, 1H, Ar-H); ^13^C-NMR: 19.9, 24.3, 25.1, 37.5, 123.6, 127.3, 127.4, 136.9, 149.8, 151.7, 164.6; C_12_H_15_N, Mol. Wt: 173.2542; Elem. Anal.: C, 83.19; H, 8.73; N, 8.08.

*2,3,3,4-Tetramethylindolenine *(**4c**): TLC: R_F_ 0.1-0.35 on silicagel eluting with CH_2_Cl_2_-CH_3_COCH_3_ (20:1); UV: (EtOH) λ_max_ (nm) 219 (H, 2.802) 259 (H, 1.007), (EtOH, HCl) 209 (H, 1.658) 235 (H, 1.234) 281 (H, 0.971); IR (cm^-1^) 2900, 1680-1700, 1570, 1420, 1350, 1220-1250, 800; ^1^H-NMR: 1.35 (s, 6H, 2×CH_3_), 2.19 (s, 3H, Ar-CH_3_), 2.37 (s, 3H, N=C-CH_3_), 6.7-7.2 (b, 3H, Ar-H); ^13^C- NMR: 18.4, 19.9, 25.4, 31.0, 119.0, 127.2, 127.3, 135.3, 142.9, 151.7, 164.6; C_12_H_15_N, Mol. Wt: 173.2542; Elem. Anal.: C, 83.19; H, 8.73; N, 8.08. 

### 4a,6-Dimethyl-1,3,3,4-tetrahydro-4aH-carbazole (**5a**)

*p*-Tolylhydrazine hydrochloride (**1a**, 0.3 g, 1.89 mmol) and 2-methylcyclohexanone (**3**, 0.21 g, 1.89 mmol) were added to glacial acetic acid (3 g, 0.05 mol). The mixture was refluxed for 1.5 h with stirring until TLC indicated the end of the reaction and formation of product. The mixture was cooled and neutralized with 1 M NaOH, then diluted with water (100 mL) and extracted with CDCl_3_ (3 × 100 mL). After drying the organic layer over Na_2_SO_4_, the solvent was removed by evaporation and the residue was passed through a short silica gel column for further purification. A red, viscous oil of 4a,6-dimethyl-1,2,3,4-tetrahydro-4a*H*-carbazole (**5a**, 0.32 g, 85%) was obtained. TLC: R_F_ 0.2 on silica gel eluting with PhCH_3_-EtOAc (9:1); UV: (EtOH) λ_max_ (nm) 212 (H, 1.624) 263 (H, 0.709), (EtOH, HCl) 204 (H, 1.080) 237 (H, 0.635), 290(H, 1.603); IR (cm^-1^): 2900, 1700, 1570, 1440, 1360, 1170, 810, 740. ^1^H-NMR: 1.15 (s, 6H, 2×CH_3_), 2.2 (s, 6H, Ar-CH_3_ and N=C-CH_3_), 1.1-2.8 (m, 8H, aliphatic ring hydrogens), 6.7-6.9 (b, 2H, Ar-H), 7.05-7.25 (b, 1H, Ar-H); ^13^C-NMR: 21.9, 22.9, 24.6, 25.4, 31.2, 34.1, 54.1, 122.0, 127.6, 131.3, 136.7, 143.4, 150.8, 187.3; C_14_H_17_N, Mol. Wt: 199.2915; Elem. Anal.: C, 84.37; H, 8.60; N, 7.03; 

### Reaction of (**1b**) with ketone (**3**) to produce a mixture of indolenines (**5b**) and (**5c**)

*m*-Tolylhydrazine hydrochloride (**1b**, 0.3 g, 1.89 mmol) and 2-methylcyclohexanone (**3**, 0.21 g, 1.89 mmol) were added to glacial acetic acid (3 g, 0.05 mol). The mixture was refluxed for 2 h with stirring until TLC indicated the end of the reaction and formation of the product. The mixture was cooled and neutralized with 1 M NaOH then diluted with water (100 mL) and extracted with CDCl_3_ (3 × 100 mL). Following drying the organic layer over Na_2_SO_4_, the solvent was removed by evaporation and the residue passed through short silica gel column for further purification. A red viscous oil that it is a mixture of the two isomers (**5b**) and (**5c**) is obtained. TLC: R_F_ 0.1-0.3 on silica gel eluting with CH_2_Cl_2_: CH_3_COCH_3_ (20: 1); UV (EtOH) λ_max_ (nm) 214 (H, 2.398) 259 (H, 0.157), (EtOH, HCl) 206 (H, 1.813) 235(H, 0.378) 281 (H, 0.150); IR (cm^-1^): 2840-2910, 1695, 1570, 1430, 1365, 1215, 1125, 965, 800, 780, 743; ^1^H-NMR: 1.14 (s, 3H), 1.1-2.8 (m, aliphatic ring), 1.24 (s, 3H), 2.21 (s, 3H), 6.4- 7.2 (m, Ar-H); ^13^C-NMR: 18.4, 21.9, 22.9, 23.2, 24.3, 25.4, 31.2, 34.1, 47.3, 53.8, 119.1, 123.7, 127.2, 127.4, 129.4, 133.6, 136.9, 137.3, 140.5, 153.7, 187.3; C_14_H_17_N, Mol. Wt: 199.2915; Elem. Anal.: C, 84.37; H, 8.60; N, 7.03.

### 4a-Methyl-6-nitro-1,2,3,4-tetrahydro-4aH-carbazole (**5e**)

*p*-Nitrophenylhydrazine (**1d**, 0.29 g, 1.89 mmol) and 2-metylcyclohexanone (**3**, 0.21 g, 1.89 mmol) were added to glacial acetic acid (3 g, 0.05 mol). The mixture was refluxed for 24 h with stirring until TLC indicated the end of the reaction and formation of product. The mixture cooled and neutralized with NaOH 1 M then diluted with water (100 mL) and extracted with CDCl_3_ (3 × 100 mL). After drying the organic layer over Na_2_SO_4_, the solvent was removed by evaporation. A yellow oil of the product (**5e**) (0.24 g, 53%) was obtained and we used a short column of silica gel for further purification. TLC: R_F_ 0.44 on silicagel eluting with PhCH_3_-CH_3_COCH_3_ (10:2); UV: (EtOH) λ_max_ (nm) 221 (H, 2.389) 304 (H, 2.413), (EtOH, HCl): 219 (H, 2.338) 302 (H, 2.370); IR: (cm^-1^) 2840-2920, 1880, 1708, 1585, 1555, 1500, 1440, 1320, 1230, 1096, 1046, 970, 830, 730; ^1^H-NMR: 1.32 (s, 3H), 1.1-2.8 (m, 8H, aliphatic ring), 7.3-7.5 (b, 1H, Ar-H), 7.85 (s, 1H, Ar-H), 7.9-8.1 (b, 1H, Ar-H); ^13^C-NMR: 21.9, 22.9, 25.4, 31.2, 34.1, 52.8, 119.6, 123.0, 124.7, 144.4, 146.7, 159.9, 187.3; C_13_H_14_N_2_O_2_; Mol. Wt. 230.2625; Elem. Anal.: C, 67.81; H, 6.13; N, 12.17; O, 13.90.

### 4a-Methyl-8-nitro-1,2,3,4-tetrahydro-4aH-cabazole (**5d**)

A mixture of *o*-nitrophenylhydrazine (**1c**, 0.29 g, 1.89 mmol), 2-methylcyclohexanone (**3**, 0.21 g, 1.89 mmol) and glacial acetic acid (3 g, 0.05 mol) was refluxed for 24 h with stirring until TLC indicated the end of the reaction. The mixture was cooled and neutralized with NaOH 1 M then diluted with water (100 mL) and extracted with CDCl_3_ (3 × 100mL). After drying the organic layer over Na_2_SO_4_, the solvent was removed by evaporation. A red oil of (**5d**) (0.23 g, 51%) was obtained and a short column of silica gel was used for further purification. TLC: R_F_ 0.2 on silica gel eluting with PhCH_3 _-CH_3_COCH_3_ (9:1); UV: (EtOH) λ_max_ (nm) 233 (H, 1.92), 251 (H, 2.03), 440 (H, 0.66) (EtOH, HCl), 233 (H, 1.87), 251 (H, 1.94), 440 (H, o.64); IR (cm^-1^): 2820-2940, 1690, 1600, 1560, 1490, 1400, 1320, 1260, 1020, 740; ^1^H-NMR: 1.3 (s, 3H), 1.1-2.8 (m, 8H, aliphatic ring) 6.5-7.8 (b, 3H, Ar-H); ^13^C-NMR: 21.9, 22.9, 25.4, 31.2, 34.1, 52.8, 119.6, 128.0, 135.6, 141.7, 143.9, 144.4, 187.3; C_13_H_14_N_2_O_2_; Mol. Wt. 230.2625; Elem. Anal.: C, 67.81; H, 6.13; N, 12.17; O, 13.90.

### 2,3,3-Trimethyl-5-Nitroindolenine (**4d**)

A mixture of *o*-nitrophenylhydrazine (**1c**, 0.29 g, 1.89 mmol), 2-methylcyclohexanone (3, 0.21 g, 1.89 mmol), and glacial acetic acid (3 g, 0.05 mol) was refluxed for 24 h with stirring until TLC indicated the end of the reaction. The mixture was cooled and neutralized with 1 M NaOH, then diluted with water (100 mL) and extracted with CDCl_3_ (3 × 100 mL). After drying the organic layer over Na_2_SO_4_, the solvent was removed by evaporation. A red oil of (**5d**) (0.23 g, 51%) was obtained and we used a short column of silica gel for additional purification. TLC: R_F_ 0.2 on silica gel eluting with PhCH_3_-CH_3_COCH_3 _(9:1); UV: (EtOH) λ_max_ (nm) 233 (H, 1.92), 251 (H, 2.03), 440 (H, 0.66): (EtOH, HCl), 233 (H, 1.87), 251 (H, 1.94), 440 (H, 0.64); IR (cm^-1^): 2820-2940, 1690, 1600, 1560, 1490, 1400, 1320, 1260, 1020, 740. ^1^H-NMR: 1.3 (s, 3H), 1.1-2.8 (m, 8H, aliphatic ring), 6.4-6.6 (b, 3H, Ar-H); ^13^C-NMR δ: 19.9, 25.1, 36.5, 119.6, 122.7, 122.9, 146.6, 153.7, 157.9, 164.6; C_11_H_12_N_2_O_2_; Mol. Wt: 204.2252; Elem. Anal.: C, 64.69; H, 5.92; N, 13.72; O, 15.67.
